# HELP-SEEKING FOR SUICIDE RISK AMONG MIDDLE- TO OLD-AGE ADULTS

**DOI:** 10.1093/geroni/igac059.2143

**Published:** 2022-12-20

**Authors:** Xiaochuan Wang, Susanny Beltran, Kim Gryglewicz

**Affiliations:** University of Central Florida, Orlando, Florida, United States; University of Central Florida, Orlando, Florida, United States; University of Central Florida, Orlando, Florida, United States

## Abstract

Suicide is a major public health concern. Suicide rates have increased steadily in the U.S. and are highest among middle-aged (ages 45-64) and older (age 75+) adults. Help-seeking represents an important coping behavior that can mitigate suicide risk. However, research on this topic is sparse, especially among older adults. To address this gap, a systematic review of the existing literature describing help-seeking for suicide risk among middle- to old-age adults was conducted. Using PRISMA guidelines, we searched electronic databases (e.g., ProQuest, EBSCOhost, PsycINFO, PubMed, Medline) and key journals with suicide and/or gerontology focuses for peer-reviewed publications in English between 2010-2020. The search yielded 4,732 unduplicated publications. After screening articles for relevance based on titles and abstracts, 52 articles were reviewed in full text. A total of 24 articles met inclusion criteria and were included in this review. The articles reviewed included a range of topics, including prevalence of service utilization, service use prior to suicide-related behaviors, and correlates of help-seeking. Overall, prevalence of service utilization was generally low and varied by suicidal history (e.g., greater prevalence among those with a history of suicide attempt, as compared to those with suicide ideation but no attempts). The systematic review also identified key service use facilitators (e.g., higher suicide literacy, previous or current suicidality) and barriers (e.g., stigma). Results of this systematic review highlight the need for future research and tailored services to improve suicide prevention and intervention strategies for middle-aged and older adults.

